# Post-Traumatic Caspase-3 Expression in the Adjacent Areas of Growth Plate Injury Site: A Morphological Study

**DOI:** 10.3390/ijms140815767

**Published:** 2013-07-29

**Authors:** Giuseppe Musumeci, Paola Castrogiovanni, Carla Loreto, Sergio Castorina, Karin Pichler, Annelie Martina Weinberg

**Affiliations:** 1 Department of Bio-Medical Sciences, Human Anatomy and Histology Section, University of Catania, Catania 95123, Italy; E-Mails: pacastro@unict.it (P.C.); carla.loreto@unict.it (C.L.); sergio.castorina@unict.it (S.C.); 2 Department of Orthopaedic Surgery, Medical University of Graz, Graz 8036, Austria; E-Mails: karin.pichler@medunigraz.at (K.P.); annelie.weinberg@t-online.de (A.W.W.)

**Keywords:** growth plate, immunohistochemistry, caspase 3, cleaved PARP-1, histological evaluation, western blot

## Abstract

The epiphyseal plate is a hyaline cartilage plate that sits between the diaphysis and the epiphysis. The objective of this study was to determine the impact of an injury in the growth plate chondrocytes through the study of histological morphology, immunohistochemistry, histomorphometry and Western Blot analyses of the caspase-3 and cleaved PARP-1, and levels of the inflammatory cytokines, Interleukin-6 (IL-6) and Tumor Necrosis Factor alpha (TNF-α), in order to acquire more information about post-injury reactions of physeal cell turnover. In our results, morphological analysis showed that in experimental bones, neo-formed bone trabeculae—resulting from bone formation repair—invaded the growth plate and reached the metaphyseal bone tissue (bone bridge), and this could result in some growth arrest. We demonstrated, by ELISA, increased expression levels of the inflammatory cytokines IL-6 and TNF-α. Immunohistochemistry, histomorphometry and Western Blot analyses of the caspase-3 and cleaved PARP-1 showed that the physeal apoptosis rate of the experimental bones was significantly higher than that of the control ones. In conclusion, we could assume that the inflammation process causes stress to chondrocytes that will die as a biological defense mechanism, and will also increase the survival of new chondrocytes for maintaining cell homeostasis. Nevertheless, the exact stimulus leading to the increased apoptosis rate, observed after injury, needs additional research to understand the possible contribution of chondrocyte apoptosis to growth disturbance.

## 1. Introduction

The epiphyseal plate is a hyaline cartilage plate that sits between the diaphysis and the epiphysis at each end of a long bone for children and adolescents. The growth plate is responsible for bone lengthening in children through endochondral ossification, involving a strictly regulated sequence of chondrocyte proliferation and maturation and extracellular matrix changes [1]. When a child’s bones have completed growing (or reached skeletal maturity), the growth plates ossify (harden) and cause the epiphysis to fuse together with the metaphysis, forming one complete bone. The hardened, mature growth plate area is then called the epiphyseal line. The growth plate has a very specific zonal arrangement, including a relatively inactive reserve zone at the epiphyseal end, moving distally into a proliferative and then hypertrophic zone and ending with a band of ossifying cartilage (the metaphysis). A mnemonic for remembering the names of the epiphyseal growth plate zones is: Resting zone, Proliferative zone, Hypertrophic cartilage zone, Calcified cartilage zone, Ossification zone [2,3]. The growth plate is clinically relevant in that, before growth is complete, the physis is susceptible to fractures because it is the most fragile region of the long bones and is prone to trauma injury. Post-traumatic overgrowth of growing long bones is a common clinical phenomenon in paediatric traumatology and is the result of an enhanced stimulation of the nearby growth plate after fracture. Due to its poor ability to regenerate itself after injury, the growth plate injury site often has structural disorganization with formation of vertical septa and a bony bridge [4–7]. When the bone bridge is large enough, the defect will result in some growth arrest, leading to angular deformities or longitudinal shortening [8]. The mechanism for bone bridge formation is not clear. Previous studies have indicated that bone bridge formation does not involve endochondral ossification [9,10], but is a result of direct intramembranous bone formation, which involves infiltration of marrow-derived fibroblast-like mesenchymal cells, bone cell differentiation, and bone matrix production [6]. Furthermore, prior to mesenchymal cell differentiation, there was an initial inflammatory response involving infiltration of inflammatory cells at the growth plate injury site [6].

With regard to inflammation, Interleukin-6 (IL-6) is a cytokine that acts as both pro- and anti-inflammatory. It is secreted by T lymphocytes and macrophages to stimulate the immune response, such as during infection or in response to a trauma or other tissue damage leading to inflammation. Moreover, osteoblasts also secrete IL-6 to stimulate the formation of osteoclasts. The Tumor Necrosis Factor alpha (TNF-α) is a cytokine involved in systemic inflammation and is a member of a group of cytokines that stimulate the acute phase reaction [11,12]. The TNF-α is involved in many processes such as apoptotic cell death, proliferation, differentiation, carcinogenesis and viral replication. The main role of TNF-α is in the regulation of cells of the immune system. Defects in the regulation, in particular the overproduction of TNF-α, are implicated in many human diseases, such as cancer [11,12].

To date, the exact post-fractural reactions of the growth plate are poorly understood and it is currently unclear what molecules of chondral cells are potentially involved in the regulation of cellular events following growth plate injury, such as programmed cell death (apoptosis). The aim of the present study is to obtain insight into the biomolecular mechanisms that underlie the histomorphological modifications on the growth plate determined by a drill hole in the left tibia proximal growth plate, as the latter simulates a traumatological injury. We hypothesized the possibility that the inflammation process causes stress to chondrocytes and induces their death as a biological defense mechanism; on the other hand, survival of new chondrocytes increases in order to maintain cell homeostasis. To develop our hypothesis, we investigated the serum levels of IL-6, TNF-α inflammatory cytokines and the morphological aspects of the growth plate through histology, the caspase-3 expression by immunohistochemistry and histomorphometry. Moreover, we conducted a Western Blot analysis to quantify the expression of caspase-3 and cleaved PARP-1.

## 2. Results and Discussion

### 2.1. Histology Observations

Histological (H & E staining) analysis of epiphyseal growth plate and time-course progression of events, in both control rats and experimental ones, was carried out.

#### Day 7

In control rats, the epiphyseal growth plate was quite wide and it was possible to distinguish its different zones. The hypertrophic zone, adjacent to the metaphysis, was quite wide, although it was not possible to show areas of bone formation in the metaphysis yet. However, epiphyseal osteogenesis was clear, with neo-formed bone trabeculae and bone marrow spaces (data not shown). The resting zone, adjacent to the epiphysis, was characterized by well-defined hyaline cartilage. The proliferating/columnar zone was present and evident too. In experimental rats, the area of the injury was first infiltrated with inflammatory cells and some cellular debris and subsequently occupied by marrow-derived fibroblast-like mesenchymal cells and characterized by hypervascularity (data not shown).

#### Day 14

**Figure 1:**
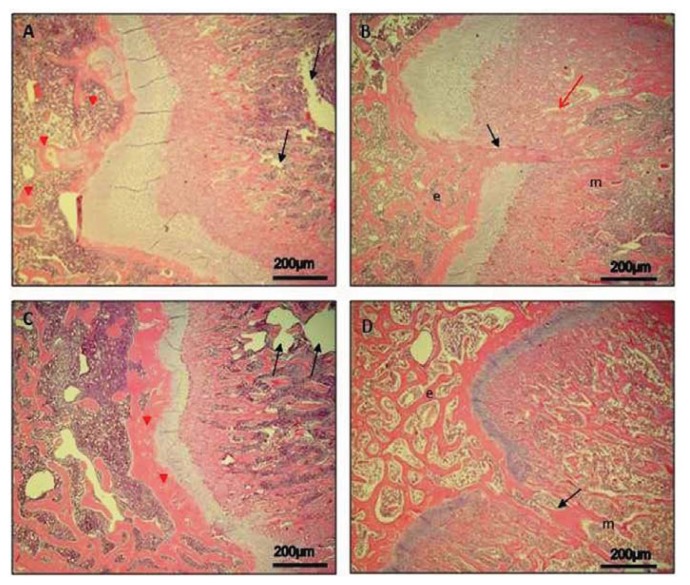
Epiphyseal growth plate in both control and experimental rats and time-course progression of osteogenesis. H&E staining. (**A**) *Day 14*, control bone: reduced epiphyseal growth plate. Metaphyseal osteogenesis (black arrows). Epiphyseal osteogenesis (red arrowheads). Magnification 10×; Scale bars: 200 μm; (**B**) *Day 14*, experimental bone: the mesenchymal cells in the bridge replaced by neo-formed bone trabeculae (black arrow) as consequences of the intramembranous osteogenesis; epiphysis (e) and metaphysis (m). Magnification 10×; Scale bars: 200 μm; (**C**) *Day 30*, control bone: considerably reduced epiphyseal growth plate. Diaphyseal osteogenesis with extension of diaphyseal marrow cavity (black arrows). Epiphyseal osteogenesis with a thick layer of bone tissue (red arrowheads). Magnification 10×; Scale bars: 200 μm; (**D**) *Day 30*, experimental bone: invasion by bone formation (black arrow), epiphysis (e) and metaphysis (m). Magnification 10×; Scale bars: 200 μm.

In control rats, the epiphyseal growth plate was reduced (Figure 1A), with a wide zone of hypertrophic cartilage, where it was also possible to observe empty lacunae and lacunae with debris of apoptotic chondrocytes. On the side of the metaphysis, the process of osteogenesis was evident with neo-formed bone trabeculae, which begin to be reabsorbed in order to allow the extension of diaphyseal marrow cavity (Figure 1A). On the side of the epiphysis, bone formation was ongoing with evident wide bone marrow spaces and well-defined bone trabeculae (Figure 1A). In experimental rats, the mesenchymal cells were reabsorbed and replaced by neo-formed bone trabeculae (Figure 1B); as consequences of the process of intramembranous osteogenesis that determines mesenchymal marrow cells differentiation into osteoprogenitor, the same cells seemed to reach and invade the neo-formed bone trabeculae on the metaphyseal side. In lateral areas, adjacent to the forming bone bridge, endochondral osteogenesis in the metaphysis is evident (Figure 1B).

#### Day 30

In control rats, the epiphyseal growth plate was considerably reduced (Figure 1C). The hypertrophic zone was also reduced, and the process of diaphyseal osteogenesis was apparently increased with further extension of diaphyseal marrow cavity (Figure 1C). On the side of the epiphysis, close to the growth plate, a thick layer of well-defined bone tissue was evident (Figure 1C). In experimental rats, the invasion by bone formation that broke, at this point, the continuity of the growth plate was evident; it reached the metaphysis where it was in continuity with the neo-formed bone tissue present there (Figure 1D). In the bone bridge, the presence of wide bone marrow spaces rich in hematopoietic tissue was clear. In lateral areas, adjacent to the bone bridge, endochondral osteogenesis in the metaphysis is evident (Figure 1D).

### 2.2. Immunohistochemistry Observations

In our results, caspase-3 immunohistochemical staining was not relevant in all analyzed days (7, 14, 30) in control rats, particularly in the growth plate; on the contrary a time-course progression of caspase-3 immunohistochemical staining was highlighted in experimental rats.

#### Day 7

**Figure 2:**
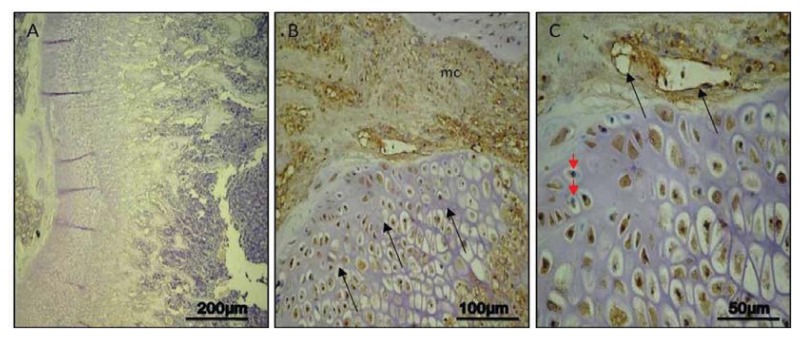
Caspase-3 immunohistochemical staining in control and experimental rats, on *Day 7*. (**A**) *Day 7*, control: no immunoreactions in the growth plate. Magnification 10×; Scale bars: 200 μm; (**B**) *Day 7*, experimental: moderate immunostaining in areas of injured growth plate (arrows) adjacent to the bridge invaded by mesenchymal cells (mc). Magnification 20×; Scale bars: 100 μm; (**C**) *Day 7*, experimental: nuclear and weakly cytoplasmic immunostaining in brown; some nuclei stained in blue (red arrowheads); ingrowth of vessels in invading mesenchymal tissue (black arrows). Magnification 40×; Scale bars: 50 μm.

In control rats, no immunoreaction in the growth plate was detected (ES:0; IS:0) (Figure 2A). In experimental rats, a moderate immunostaining (ES:++; IS:2) was shown in areas of injured growth plate, adjacent to the bridge invaded by mesenchymal cells; in immunostained chondrocytes, it was not always possible to distinguish the nucleus and immunostaining was often widespread in the lacunae. Immunoreaction was also detected in invading mesenchymal tissue where a healing process was ongoing (Figure 2B). At higher magnification, nuclear and weakly cytoplasmic immunostaining was clear (brown) though some nuclei were stained in blue by haematoxylin; ingrowth of vessels could be observed in invading mesenchymal tissue (Figure 2C).

#### Day 14

**Figure 3:**
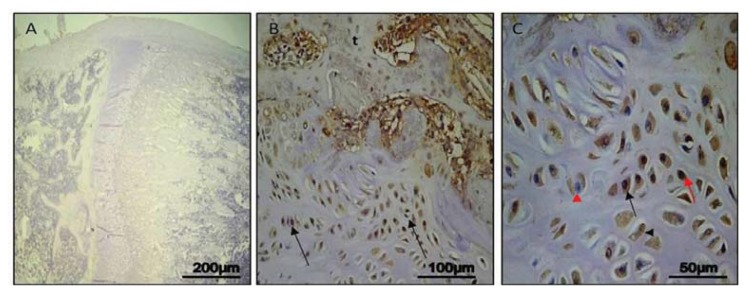
Caspase-3 immunohistochemical staining in control and experimental rats, on *Day 14*. (**A**) *Day 14*, control: no immunostaining in the growth plate. Magnification 10×; Scale bars: 200 μm; (**B**) *Day 14*, experimental: strong immunostaining in chondrocytes (arrows) close to neo-trabeculae (t) of forming bone bridge. Magnification 20×; Scale bars: 100 μm; (**C**) *Day 14*, experimental: cytoplasmic (black arrow) or nuclear (red arrow) immunostaining of chondrocytes; non immunostained nuclei (red arrowhead); lacunae with immunostained debris (black arrowhead). Magnification 40×; Scale bars: 50 μm.

In control rats, no immunostaining in the growth plate was detected (ES:0; IS:0) (Figure 3A). In experimental rats, a strong immunostaining (ES:+++; IS:3) was evident in hypertrophic chondrocytes, close to neo-trabeculae of the forming bone bridge. Immunostaining was mainly cytoplasmic; in many chondrocytes the nucleus was immunostained too, in others it was stained in blue by haematoxylin. Lacunae with immunostained debris were also present. Immunostaining could be observed in areas of bone formation (Figure 3B). At higher magnification, it was possible to better appreciate the cytoplasmic, or nuclear, immunostaining of chondrocytes, the presence of non immunostained nuclei, and lacunae with immunostained debris, as evidence of the ongoing apoptotic process in all its stages (Figure 3C).

#### Day 30

**Figure 4:**
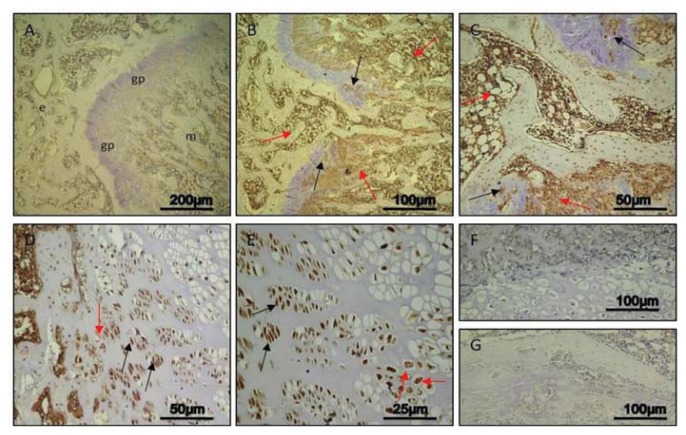
Caspase-3 immunohistochemical staining in control and experimental rats, on *Day 30*. (**A**) *Day 30*, control: weak immunoreaction in areas of metaphyseal (m), epiphyseal (e), and bone formation; no immunostaining in growth plate (gp). Magnification 10×; Scale bars: 200 μm; (**B** and **C**) *Day 30*, experimental: very strong immunostaining in areas of bone formation (red arrows) and in areas of injured growth plate strictly close to the bone bridge (black arrows); (**B**) Magnification 20×; Scale bars: 100 μm; (**C**) Magnification 40×; Scale bars: 50 μm; (**D** and **E**) *Day 30*, experimental: very strong immunostaining at cytoplasmic and nuclear level in chondrocytes (black arrows); immunostained debris (red arrows) in lacunae; (**D**) Magnification 40×; Scale bars: 50 μm; (**E**) Magnification 60×; Scale bars: 25 μm; (**F**) *Day 30*, control: immunostaining negative control. No immunostaining detected. Magnification 20×; Scale bars: 100 μm; (**G**) *Day 30*, experimental: immunostaining negative control. No immunostaining observed. Magnification 20×; Scale bars: 100 μm.

**Figure 5:**
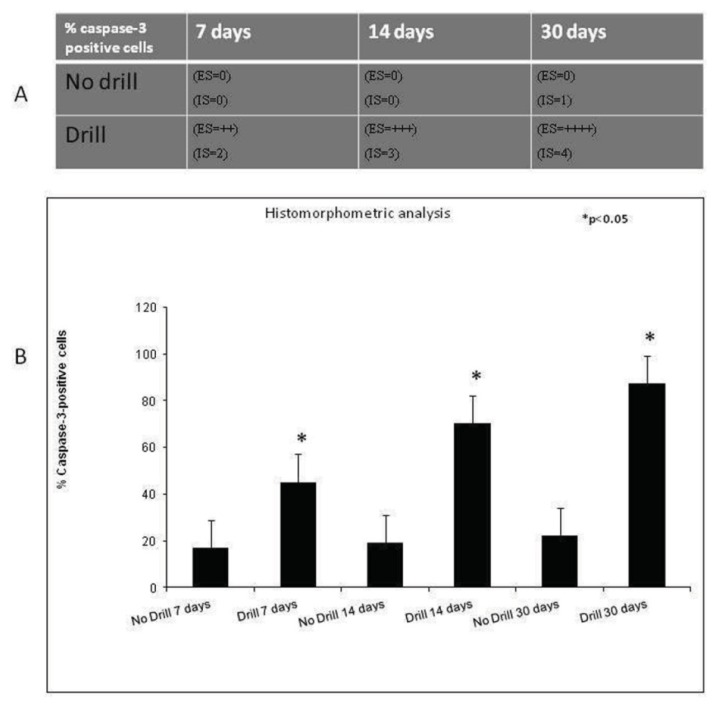
(**A**) Immunohistochemical grading. Intensity of staining (IS) was graded on a scale of 0–4, according to the following assessment: no detectable staining (0), weak staining (1), moderate staining (2), strong staining (3), very strong staining (4). The percentages of Caspase-3 immunopositive cells (Extent Score = ES) were scored as a percentage of the final number of 100 cells in five categories: <5% (0); 5%–30% (+); 31%–50% (++); 51%–75% (+++), and >75% (++++); (**B**) Histomorphometric analysis. Percentage of Caspase-3 positive cells out of the total number of cells counted in fractured growing bones. Results are presented as the mean ± SEM. ANOVA and Bonferroni’s test, were used to evaluate the significance of the results. * *p* < 0.05, when compared to the unfractured growing bones.

In control rats, only a weak immunoreaction was observed (ES:0; IS:1) in areas of metaphyseal and epiphyseal bone formation, whereas no immunostaining was detected in growth plate (Figure 4A). In experimental rats, a very strong immunostaining (ES:++++; IS:4) was shown not only in areas of bone formation, but also in areas of injured growth plate very close to the bone bridge (Figure 4B,C). At higher magnification, the evidently very strong immunostaining was at cytoplasmic and nuclear levels in chondrocytes, and it also involved debris in lacunae (Figure 4D,E). No immunostaining was observed in the negative control carried out both in control rats (Figure 4F) and experimental ones (Figure 4G). In all analyzed different time points (7, 14, 30 days), both the percentage of caspase-3 positive cells and the IS was significantly greater (*p* < 0.05) in experimental rats compared to control ones (Figure 5A).

### 2.3. Histomorphometric Analysis

**Figure 6:**
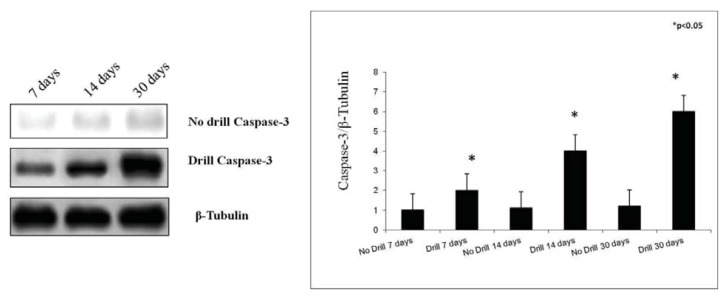
Caspase-3 expression in control and in experimental growth plate chondrocytes determined by Western Blot analysis. Data show the relative expression (mean ± SEM) of caspase-3 calculated as arbitrary densitometric units (A.D.U.), collected from three independent experiments. * *p* < 0.05 compared to control. Representative immunoblots were obtained using 50 μg of cell homogenates for each experimental group. β-Tubulin was used as loading control in each experiment.

Caspase-3 expression was highlighted in areas of metaphyseal and epiphyseal bone formation both in control and in experimental rats, in growth plate areas adjacent to the injury site close to the forming bone bridge and in the injury site where an osteogenic process was taking place. We found significant differences between control and experimental physis areas, at different time points (7, 14, 30 days). The immunohistochemical analysis showed that the Caspase-3 was localized in different areas of bone physis and in different cells as well as in cellular debris where present (Figure 6B).

### 2.4. Western Blot Analysis

**Figure 7:**
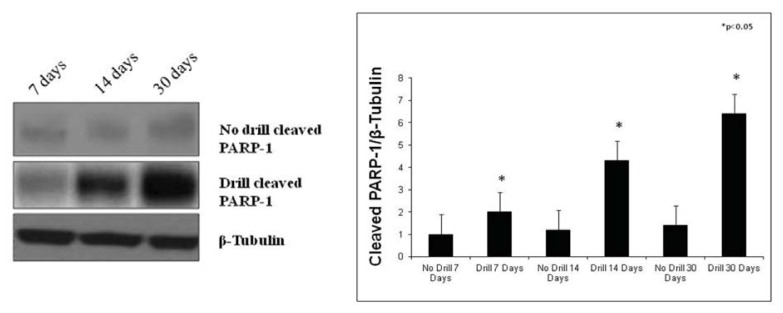
Cleaved PARP-1 expression in control and in experimental growth plate chondrocytes determined by Western Blot analysis. Data show the relative expression (mean ± SEM) of cleaved PARP-1 calculated as arbitrary densitometric units (A.D.U.), collected from three independent experiments. * *p* < 0.05 compared to control. Representative immunoblots were obtained using 50 μg of cell homogenates for each experimental group. β-Tubulin was used as loading control in each experiment.

In this study, we also examined caspase-3 and cleaved PARP-1 protein expression in the growth plate chondrocytes by Western Blot analysis in control and in experimental rats after the sacrifice. Data obtained from these analyses showed caspase-3 and cleaved PARP-1 expressions were stronger in experimental rats, while in control they were weak. More specifically, caspase-3 and cleaved PARP-1 protein expression was increased over a time-course progression (Figures 6 and 7).

### 2.5. Biochemical Studies

**Figure 8:**
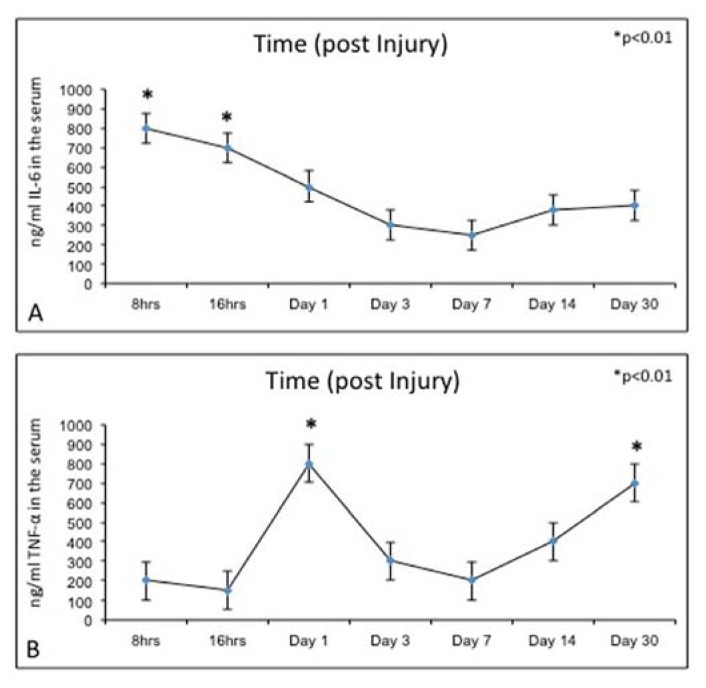
(**A**) ELISA: IL-6 levels, at time progression post injury, were quantified in experimental rats. Results are presented as the mean ± SEM. ANOVA and Bonferroni’s test were used to evaluate the significance of the results: * *p* < 0.01, considered highly statistically significant; (**B**) ELISA: TNF-α levels, at time progression post injury, were quantified in experimental rats. Results are presented as the mean ± SEM. ANOVA and Bonferroni’s test were used to evaluate the significance of the results: * *p* < 0.01, considered highly statistically significant.

In this study, we examined also the serum cytokine (IL-6, TNF-α) levels in control and experimental rats by means of the ELISA test. IL-6 and TNF-α serum levels were significantly lower in control rats (data not shown). However, experimental rats showed higher serum levels of IL-6 (Figure 8A) and TNF-α (Figure 8B). In particular, the IL-6 and TNF-α level observed within 24 h after growth plate damage was drastically increased, coinciding with the initial acute transient inflammatory phase. Meanwhile, during the time progression from day 1 to day 30, the IL-6 level decreased (Figure 8A). Conversely, during the time progression from day 1 to day 30, the TNF-α level increased (Figure 8B).

The growth plate is the most fragile area of the growing long bones in children; because bone fracture in children is a common event and it may result in growth plate damage, this can represent a significant problem for the developing long bone, particularly in a young child who has a significant remaining growth period [13]. Since growth plate cartilage has limited ability to regenerate itself, injury can represent a significant problem for the developing long bone. It depends on the level at which the injury occurs in the physis [5,14]. After a bone fracture, bone repair is a highly specialized process of wound healing that involves complex cellular and molecular events associated with both endochondral and intramembranous ossification. Interaction of different cell types (such as inflammatory cells, mesenchymal cells, chondrocytes, endothelial cells, osteoblasts, and osteoclasts) and molecules (such as cytokines, growth factors, and matrix proteins) [15] restore the bone to its original structural integrity [16]. Growth plate injury-induced bone bridge formation and subsequently, bone growth arrest are a significant orthopedic problem and although some studies have attempted to explore therapeutic strategies for growth plate injury repair in animal models, there are relatively few studies that have tried to investigate the cellular and molecular mechanisms underlying the injury-induced bone bridge formation at the growth plate injury site. After fracture, while the damaged bone heals and restores to its original structure, the repair mechanism of the injured growth plate often results in the formation of bone rather than the original cartilage structure at the injury site, which may lead to orthopedic problems such as limb length discrepancy and bone angulations deformity. A previous study using a rat growth plate drill-hole injury model showed that the formation of a bone bridge extending from the epiphysis to the metaphysis of the proximal tibia was via the intramembranous ossification mechanism and did not involve endochondral ossification [6]. Bone bridge is a result of direct bone formation from osteoblasts differentiated from infiltrating marrow-derived mesenchymal cells [6].

As in bone fracture and soft tissue injury, the first stage of cellular response to growth plate trauma is the inflammatory response where inflammation infiltrates at the injury site secrete cytokines and growth factors that may be important regulators of subsequent healing responses. Proinflammatory cytokines, IL-6 and TNF-α, are involved in regulating the inflammatory response to tissue injury or bone fracture [11]. These findings suggest IL-6 and TNF-α may play important regulatory roles during the initial inflammatory response to growth plate injury. Since the inflammatory phase is the first stage of injury response that may play important roles in controlling the subsequent healing events, IL-6 and TNF-α are known to have inhibitory effects on gene expression of cartilage matrix proteins and have apoptotic effects on chondrocytes [12].

In our study, we demonstrated that in control bones morphological analysis of the time-course progression of osteogenesis showed that it took place normally. In the experimental bones, the morphological analysis showed how the area of the drill-hole injury was first filled by inflammatory cells and some cellular debris, later occupied by marrow-derived fibroblast-like mesenchymal cells characterized by hypervascularity. Subsequently, mesenchymal cells were reabsorbed and completely replaced by bone trabeculae, probably resulting from activation of mesenchymal marrow cells differentiating into osteoprogenitor cells. Neo-formed bone trabeculae invaded the growth plate and reached the metaphyseal bone bridge.

Morphological analysis of our study supports literature data [2,4,6,7,14–18]. When the bone bridge is large enough, the defect will result in some growth arrest, leading to angular deformities or longitudinal shortening [8]. For this reason it is important to never underestimate a contusion or a distortion to the limbs before skeletal development is completed since it can result in permanent injury.

It is instead less clear what happens at the adjacent uninjured area of the growth plate. Some investigators examined the effects of injury on the growth plate structure and composition, and the cellular and molecular changes at the injury site and at the adjacent uninjured area. Interestingly, their immunohistochemical analyses revealed reduced chondrocyte proliferation but increased apoptosis in the adjacent uninjured area [7]. The two major mechanisms regulating apoptosis include: the intrinsic pathway mediated by mitochondria and the extrinsic pathway induced by death signaling ligands that induce caspase-8 initiator protease, which activates executioner proteases such as caspase-3 [19–23]. Caspases are a group of intracellular cysteine enzymes that destroy essential cellular proteins, leading to controlled cell death. There are two tiers of caspase activation during apoptosis. Initiator caspases (caspases 2, 8, 9, and 10) are activated through the apoptosis-signaling pathways and activate the effector caspases (caspases 3, 6, and 7), which, in an expanding cascade, carry out apoptosis [24,25]. Effector caspases play a vital role in the induction, transduction, amplification, and execution of apoptotic signals within the cell [26]. PARP-1 is a nuclear DNA repair enzyme that is implicated in DNA repair and maintenance of genomic integrity [27]. Caspase-mediated processing of PARP-1 effectively results in potent and irreversible inactivation of the protein. In apoptosis, PARP-1 is cleaved by activated caspase-3 between Asp214 and Gly215, resulting in the formation of an *N*-terminal 24 kDa fragment containing most of the DNA binding domain and a *C*-terminal 89 kDa fragment containing the catalytic domain [28,29]. Inactivation of PARP-1 will result in the prevention of NAD+ depletion caused by PARP-1 activation. So, the proteolysis of PARP-1 renders the enzyme inactive and this further facilitates apoptotic cell death. Thus, the presence of 89 kDa PARP-1 fragment is considered to be an important biomarker of apoptosis [27].

In our study, we analyzed caspase-3 and cleaved PARP-1 expression in areas of the growth plate adjacent to the drill hole to determine if the injury has an effect on chondrocytes apoptosis. A time-course progression of caspase-3 immunoreaction was observed, *in vivo*, in experimental rats. From day 7–30 post-injury, caspase-3 expression increased in chondrocytes of adjacent areas of growth plate injury site, close to the forming bone bridge, and in cartilage lacunae in which cellular debris were present, as evidence of an ongoing apoptotic process. The apoptosis percentage of physeal chondrocytes was statistically compared among experimental and control bones. The physeal apoptosis rate of the experimental bones was significantly higher than that of the control ones. These data were also strengthened by Western Blot analysis, *in vitro*, where caspase-3 and cleaved PARP-1 expression significantly increased in growth plate chondrocytes with a time-course progression in experimental rats. Although we used apoptotic markers, such as caspase-3 and cleaved PARP-1, different from those used by other researchers, our study supports data from literature [2,7]. In the current study, we also investigated the expression of IL-6 and TNF-α serum levels within 24 h after growth plate damage, coinciding with the initial acute transient inflammatory phase, which was accompanied by a large influx of immune cells to the injury site. In particular, the IL-6 and TNF-α level observed within 24 h after growth plate damage were drastically increased, while during the time progression from day 1 to day 30, the IL-6 level decreased. Conversely, during the time progression from day 1 to day 30, the TNF-α level increased, probably because TNF is involved in numerous processes such as apoptotic cell death.

## 3. Experimental Section

### 3.1. Animal Studies

All animal experiments were approved by the ethical board of the Austrian Federal Ministry of Science and Research (file number: BMBWK-66.010/0054-BrGT/2006). Sprague-Dawley- Rats (4 weeks old, 110–140 g) were used to establish Caspase-3, cleaved PARP-1, IL-6 and TNF-α expression/levels in the growth plate of experimental and control growing bones. The age of the rats was chosen to ensure the maximum time of longitudinal bone growth, as growth slows by 10 weeks of age. Twenty-two animals, purchased from the Division for Laboratory Animal Science and Genetics in Himberg (Medical University of Vienna, Core Unit for Biomedical Research, Vienna, Austria), were randomly divided into two groups, experimental and control groups. Euthanasia was at 7, 14, and 30 days. Control groups consisted of control rats, that provided right and left tibia as control samples, considered to be equivalent. All rats were housed in pairs with a light to dark cycle of 12:12 h. Experimental rats sustained a 1.2 mm drill hole in the left tibia proximal growth plate under general anaesthesia (intra-muscular injections of Ketanest-S^®^ (90 mg/kg) and Rompun^®^ (5 mg/kg)). Postoperatively rats were returned to their litters for nursing and subsequent ad libitum activity. Analgesia was provided to all rats by subcutaneous injection of a painkiller (Rimadyl^®^ 4–5 mg/kg daily for one week) which was followed by addition of Novalgin^®^-drops to drinking water; control groups also received painkillers to simulate the same operating conditions of the experimental rats even if the control rats did not undergo the drill hole. After euthanasia, performed at 7, 14 and 30 days post-injury by intracardial Pentothal^®^ injection under Furane^®^-narcosis, the tibiae from both experimental and control rats were harvested and dissected from soft tissue. These were then used to perform histological, histomorphometric, immunohistochemical and Western Blot evaluations as previously described [30] and for the cytokines serum levels we used the ELISA method. In no case did the fracture callus extend into the growth plate. The time points were selected according to different phases of drill-hole injury healing, *i.e.*, after the acute inflammatory phase when the injury was filled with marrow-derived fibroblast-like mesenchymal cells (day 7), when the drill-hole injury was bridged by bony trabeculae that confer weight-bearing properties on the experimental bone (day 14) and at the time of stable bone tissue formation (1 month).

### 3.2. Histology

The tibiae were explanted and cleaned of soft tissues and fixed in 10% buffered-formalin and decalcified in 12.5% ethylenediaminetetraacetic acid (EDTA) (Sigma-Aldrich, Milan, Italy) + 1.25% sodium hydroxide (NAOH) (Sigma-Aldrich, Milan, Italy) for approximately 3 weeks. The endpoint was determined by X-ray. Following overnight wash, specimens were treated as previously described [31]. Frontal sections of tibiae were used for histological analysis. Sections 4–5 μm in thickness were obtained according to routine procedures, mounted on sialane-coated slides and stored at room temperature. Slides were dewaxed in xylene, hydrated using graded ethanol, and stained for routine histologic evaluation by Haematoxylin and Eosin (Histolab Products AB, Goteborg, Sweden) for the morphological structure as previously described [32]. The sections were observed with an Axioplan Zeiss light microscope (Jena, Germany) and photographed with a digital camera a digital camera (AxioCam MRc5, Carl Zeiss, Oberkochen, Germany).

### 3.3. Immunohistochemistry

Specimens, following overnight wash, were dehydrated in graded ethanol, cleared in xylene and paraffin-embedded, with their anatomical orientation preserved. Sections were processed as previously described [33,34]. Briefly they were incubated for 30 min in 0.3% H_2_O_2_/methanol to quench endogenous peroxidase activity then rinsed for 20 min with phosphate-buffered saline (PBS; Bio-Optica, Milan, Italy). The sections were heated (5 min × 3) in capped polypropylene slide-holders with citrate buffer (10 mM citric acid, 0.05% Tween 20, pH 6.0; Bio-Optica, Milan, Italy), using a microwave oven (750 W) to unmask antigenic sites. The blocking step was performed before application of the primary antibody with goat serum (Vector Laboraties, Chicago, IL, USA), 1:20 work dilution in PBS-T, 1 h in a moist chamber. Then, the sections were incubated overnight at 4 °C with antibodies: rabbit monoclonal Anti-Caspase-3, work dilution in PBS 1:200 (ab32351, abcam, Cambridge, UK). The secondary antibody, biotinylated anti-mouse antibody was applied for 30 min at room temperature, followed by the avidin-biotin-peroxidase complex (Vector Laboraties, Chicago, IL, USA) for a further 30 min at room temperature. The immunoreaction was visualized by incubating the sections for 4 min in a 0.1% 3,3′-diaminobenzidine and 0.02% hydrogen peroxide solution (DAB substrate kit, Vector Laboraties, Chicago, IL, USA). The sections were lightly counterstained with Mayer’s Haematoxylin (Histolab Products AB, Goteborg, Sweden) mounted in GVA mount (Zymed, Laboratories Inc., San Francisco, CA, USA) and observed with an Axioplan Zeiss light microscope (Jena, Germany).

### 3.4. Evaluation of Immunohistochemistry

The Caspase-3 staining status was identified as either negative or positive. Immunohistochemistry positive staining was defined as the presence of brown chromogen detection on the edge of the hematoxylin-stained cell nucleus, distributed within the cytoplasm or in the membrane via evaluation by light microscopy as previously described [35,36]. Stain intensity and the proportion of immunopositive cells were also assessed by light microscopy. Intensity of staining (IS) was graded on a scale of 0–4, according to the following assessment: no detectable staining (0), weak staining (1), moderate staining (2), strong staining (3), very strong staining (4). The percentage of Caspase-3 immunopositive cells (Extent Score = ES) was independently evaluated by 3 investigators (2 anatomical morphologists and one histologist) and scored as a percentage of the final number of 100 cells in five categories: <5% (0); 5%–30% (+); 31%–50% (++); 51%–75% (+++), and >75% (++++). Counting was performed at 200× magnification. Positive and negative controls were performed to test the specific reaction of primary antibodies used in this study at a protein level. Positive control testing involved sections that underwent an immunoperoxidase process, cervical carcinoma tissue. Positive immunolabeling for antibodies were membrane/cytoplasmic. Negative controls used sections of bone treated with normal rabbit serum instead of specific antibodies.

### 3.5. Computerized Morphometry Measurements and Image Analysis

Fifteen fields randomly selected from each section were analyzed and the percent area stained with Caspase-3 antibody was calculated using an image analyzer (Image-Pro Plus 4.5.1, Immagini & Computer, Milan, Italy), which quantifies the level of positive immunolabelling in each field, as described previously [37,38]. Digital pictures were taken using the Axioplan Zeiss light microscope and photographed using the Canon digital camera; Evaluations were made by four blinded investigators, whose evaluations were assumed to be correct if values were not significantly different. In case of disputes concerning the interpretation, the case has been revised to reach a unanimous agreement.

### 3.6. Western Blot Analysis

Crude extracts were prepared by homogenizing growth plate chondrocytes in a buffer containing 20 mM Tris (pH 7.4), 2 mM EDTA, 0.5 mM EGTA; 50 mM mercaptoethanol, 0.32 mM sucrose and a protease inhibitor cocktail (Roche Diagnostics, Indianapolis, IN, USA) using a Teflon-glass homogenizer and then sonicated. Protein concentrations were determined by Bradford’s method [39] using BSA as a standard. Sample proteins (50 μg) were diluted in 4× sodium dodecyl sulphate (SDS) protein gel loading solution (Invitrogen, Monza, Italy), boiled for 5 min, separated on 4%–12% Bis-tris gel (Invitrogen) and electroblotted to nitrocellulose membrane (Invitrogen, Monza, Italy). Nonspecific binding was blocked for 2 h at 37 °C with 10% nonfat dry milk in Tween-Tris-buffered saline (TTBS). Membranes were incubated overnight at 4 °C with the following antibodies: rabbit monoclonal Anti-Caspase-3 antibody (Abcam, ab32351, 1:5000 working dilution), molecular weight 32 kDa, mouse monoclonal anti-cleaved PARP-1 antibody (Santa Cruz Biotechnology Inc., Milan, Italy) sc-56196, 1:1000 working dilution) molecular weight 89 kDa, and rabbit anti-β-tubulin (Santa Cruz Biotechnology Inc., sc-9104, 1:200 working dilution) molecular weight 48 kDa, which was used as loading control. Secondary antibodies (GE Healthcare, Milan, Italy) were diluited at 1:10,000 for all antibodies. All antibodies were prepared in 10% nonfat dry milk solution in TTBS. Blots were developed using enhanced chemiluminescence ECL technique (GE Healthcare, Milan, Italy) and relative band densities were quantified using ImageQuantTL software7.0 (GE Healthcare, Milan, Italy). No signal was detected when the primary antibody was omitted (data not shown).

### 3.7. Biochemical Studies

Concentrations of Interleukin-6 (IL-6) and Tumor Necrosis Factor alpha (TNF-α) in the serum of control and experimental rats were measured using a commercially available ELISA kit (Thermo Scientific Rat IL-6 ELISA Kit, USA and Thermo Scientific Rat TNF alpha ELISA Kit, Milan, Italy), according to the manufacturer’s instructions for the quantitative levels in rat serum determination of IL-6 and TNF-α.

### 3.8. Statistical Analysis

Statistical analysis was performed using SPSS software (SPSS^®^ release 16.0, Chicago, IL, USA). Data were tested for normality with the Kolmogorov-Smirnov test. All variables were normally distributed. Comparisons between two means were tested with the Student’s *t* test, whilst comparisons between more than two groups were tested using analysis of variance (ANOVA) and Bonferroni’s test. *p*-values of less than 0.05 were considered statistically significant and *p*-values of less than 0.01 were highly statistically significant. Data are presented as the mean ± SEM. Cohen’s kappa was applied, as previously described [40,41].

## 4. Conclusions

In conclusion, we postulated that the inflammation process causes stress to chondrocytes that will die as a biological defense mechanism, and also increases the survival of new chondrocytes for maintaining cell homeostasis. In our study, the morphological analysis clearly showed how the area of the drill-hole injury was invaded by the bone bridge. Based on our hypothesis that the inflammatory process, demonstrated by the high cytokines levels, contributes to the activation of the apoptosis mechanisms, we have established by means of histomorphometric, immunohistochemical and Western Blot evaluations that the chondrocyte apoptosis rate was significantly higher in the experimental rats. Our preliminary work should improve knowledge of the effect of post-injury reactions on the growth plate. Nevertheless, the exact stimulus leading to the increased apoptosis rate, observed after injury, needs additional research to understand the possible contribution of chondrocyte apoptosis to growth disturbance.

## References

[1] Iannotti J.P. (1990). Growth plate physiology and pathology. Orthop. Clin. North. Am.

[2] Gaber S., Fischerauer E.E., Fröhlich E., Janezic G., Amerstorfer F., Weinberg A.M. (2009). Chondrocyte apoptosis enhanced at the growth plate: A physeal response to a diaphyseal fracture. Cell Tissue Res.

[3] Mirza R., Qiao S., Tateyama K., Miyamoto T., Xiuli L., Seo H. (2012). 3β-Hydroxysterol-Delta24 reductase plays an important role in long bone growth by protecting chondrocytes from reactive oxygen species. J. Bone Miner. Metab.

[4] Bailey R.W., Dubow H.I. (1981). Evolution of the concept of an extensible nail accommodating to normal longitudinal bone growth. Clin. Orthop.

[5] Wattenbarger J.M., Gruber H.E., Phieffer L.S. (2002). Physeal fractures, part I: Histologic features of bone, cartilage, and bar formation in a small animal model. J. Pediatr. Orthop.

[6] Xian C.J., Zhou F.H., McCarty R.C., Foster B.K. (2004). Intramembranous ossification mechanism for bone bridge formation at the growth plate cartilage injury site. J. Orthop. Res.

[7] Macsai C.E., Hopwood B., Chung R., Foster B.K., Xian C.J. (2011). Structural and molecular analyses of bone bridge formation within the growth plate injury site and cartilage degeneration at the adjacent uninjured area. Bone.

[8] Ogden J.A. Skeletal Injury in the Child.

[9] Lee M.A., Nissen T.P., Otsuka N.Y. (2000). Utilization of a murine model to investigate the molecular process of transphyseal bone formation. J. Pediatr. Orthop.

[10] Nagai H., Aoki M. (2002). Inhibition of growth plate angiogenesis and endochondral ossification with diminished expression of MMP-13 in hypertrophic chondrocytes in FGF-2-treated rats. J. Bone Miner. Metab.

[11] Kon T., Cho T.J., Aizawa T., Yamazaki M., Nooh N., Graves D., Gerstenfeld L.C., Einhorn T.A. (2001). Expression of osteoprotegerin, receptor activator of NF-kappaB ligand (osteoprotegerin ligand) and related proinflammatory cytokines during fracture healing. J. Bone Miner. Res.

[12] Aizawa T., Kon T., Einhorn T.A., Gerstenfeld L.C. (2001). Induction of apoptosis in chondrocytes by tumor necrosis factor-alpha. J. Orthop. Res.

[13] Foster B.K., Johnstone E.W., Benson M., Fixsen J., MaCnicol M., Parsch K. (2000). Management of Growth Plate Injuries. Paediatric Orthopaedics and Fractures.

[14] Brashear H.R. (1959). Epiphyseal fractures: A microscopic study of the healing process in rats. J. Bone Joint Surg.

[15] Zhou F.H., Foster B.K., Sander G., Xian C.J. (2004). Expression of proinflammatory cytokines and growth factors at the injured growth plate cartilage in young rats. Bone.

[16] Joyce M.E., Jingushi S., Scully S.P., Bolander M.E. (1991). Role of growth factors in fracture healing. Prog. Clin. Biol. Res.

[17] Xian C.J. (2007). Roles of epidermal growth factor family in the regulation of postnatal somatic growth. Endocr. Rev.

[18] Pichler K., Loreto C., Leonardi R., Reuber T., Weinberg A.M., Musumeci G. (2013). In rat with glucocorticoid-induced osteoporosis, RANKL is downregulated in bone cells by physical activity (treadmill and vibration stimulation training). Histol. Histopathol.

[19] Caltabiano R., Leonardi R., Musumeci G., Bartoloni G., Rusu M.C., Almeida L.E., Loreto C. (2013). Apoptosis in temporomandibular joint disc with internal derangement involves mitochondrial-dependent pathways. An *in vivo* study. Acta Odontol. Scand.

[20] Loreto C., Musumeci G., Castorina A., Loreto C., Martines G. (2011). Degenerative disc disease of herniated intervertebral discs is associated with extracellular matrix remodelling, vimentin-positive cells and cell death. Ann. Anat.

[21] Loreto C., Musumeci G., Leonardi R. (2009). Chondrocyte-like apoptosis in temporomandibular joint disc internal derangement as a repair-limiting mechanism. An *in vivo* study. Histol. Histopathol.

[22] Cardile V., Musumeci G., Sicurezza E., Caggia S., Rusu M.C., Leonardi R., Loreto C. (2013). TRAIL and its receptors DR5 and DcR2 expression, in Orthodontic Tooth Movement. Histol. Histopathol.

[23] Musumeci G., Loreto C., Leonardi R., Castorina S., Giunta S., Carnazza M.L., Trovato F.M., Pichler K., Weinberg A.M. (2013). The effects of physical activity on apoptosis and lubricin expression in articular cartilage in rats with glucocorticoid-induced osteoporosis. J. Bone Miner. Metab.

[24] Musumeci G., Loreto C., Carnazza M.L., Martinez G. (2011). Characterization of apoptosis in articular cartilage derived from the knee joints of patients with osteoarthritis. Knee Surg. Sports Traumatol. Arthrosc.

[25] Loreto C., Barbagli G., Djinovic R., Vespasiani G., Carnazza M.L., Miano R., Musumeci G., Sansalone S. (2011). Tumor necrosis factor-related apoptosis-inducing ligand (TRAIL) and its death receptor (DR5) in Peyronie’s disease. A biomolecular study of apoptosis activation. J. Sex. Med..

[26] Musumeci G., Loreto C., Carnazza M.L., Strehin I., Elisseeff J. (2011). OA cartilage derived chondrocytes encapsulated in poly(ethylene glycol) diacrylate (PEGDA) for the evaluation of cartilage restoration and apoptosis in an *in vitro* model. Histol. Histopathol.

[27] Virág L., Szabó C. (2002). The therapeutic potential of poly(ADP-ribose) polymerase inhibitors. Pharmacol. Rev.

[28] Shakibaei M., John T., Seifarth C., Mobasheri A. (2007). Resveratrol inhibits IL-1 beta-induced stimulation of caspase-3 and cleavage of PARP in human articular chondrocytes *in vitro*. Ann. N. Y. Acad. Sci.

[29] Shakibaei M., Csaki C., Nebrich S., Mobasheri A. (2008). Resveratrol suppresses interleukin-1beta-induced inflammatory signaling and apoptosis in human articular chondrocytes: Potential for use as a novel nutraceutical for the treatment of osteoarthritis. Biochem. Pharmacol.

[30] Musumeci G., Trovato F.M., Imbesi R., Castrogiovanni P. (2013). Effects of dietary extra-virgin olive oil on oxidative stress resulting from exhaustive exercise in rat skeletal muscle: A morphological study. Acta Histochem.

[31] Castrogiovanni P., Musumeci G., Trovato F.M., Avola R., Magro G., Imbesi R. (2013). Effects of high-tryptophan diet on pre- and postnatal development in rats: A morphological study. Eur. J. Nutr..

[32] Musumeci G., Carnazza M.L., Loreto C., Leonardi R., Loreto C. (2012). β-Defensin-4 (HBD-4) is expressed in chondrocytes derived from normal and osteoarthritic cartilage encapsulated in PEGDA scaffold. Acta Histochem.

[33] Musumeci G., Lo Furno D., Loreto C., Giuffrida R., Caggia S., Leonardi R., Cardile V. (2011). Mesenchymal stem cells from adipose tissue which have been differentiated into chondrocytes in three-dimensional culture express lubricin. Exp. Biol. Med.

[34] Musumeci G., Loreto C., Carnazza M.L., Coppolino F., Cardile V., Leonardi R. (2011). Lubricin is expressed in chondrocytes derived from osteoarthritic cartilage encapsulated in poly (ethylene glycol) diacrylate scaffold. Eur. J. Histochem.

[35] Leonardi R., Loreto C., Talic N., Caltabiano R., Musumeci G. (2012). Immunolocalization of lubricin in the rat periodontal ligament during experimental tooth movement. Acta Histochem.

[36] Musumeci G., Carnazza M.L., Leonardi R., Loreto C. (2012). Expression of β-Defensin-4 in “an *in vivo* and *ex vivo* model” of human osteoarthritic knee meniscus. Knee Surg. Sports Traumatol. Arthrosc.

[37] Musumeci G., Loreto C., Clementi G., Fiore C.E., Martinez G. (2011). An *in vivo* experimental study on osteopenia in diabetic rats. Acta Histochem.

[38] Leonardi R., Rusu M.C., Loreto F., Loreto C., Musumeci G. (2012). Immunolocalization and expression of lubricin in the bilaminar zone of the human temporomandibular joint disc. Acta Histochem..

[39] Bradford M.M. (1976). A rapid and sensitive method for the quantitation of microgram quantities of protein utilizing the principle of protein–dye binding. Anal. Biochem.

[40] Musumeci G., Loreto C., Carnazza M.L., Cardile V., Leonardi R. (2013). Acute injury affects lubricin expression in knee menisci. An immunohistochemical study. Ann. Anat.

[41] Musumeci G., Leonardi R., Carnazza M.L., Cardile V., Pichler K., Weinberg A.M., Loreto C. (2013). Aquaporin 1 (AQP1) expression in experimentally induced osteoarthritic knee menisci: An *in vivo* and *in vitro* study. Tissue Cell.

